# Making a Case for Creating Living Labs for Aging-in-Place: Enabling Socially Innovative Models for Experimentation and Complementary Economies

**DOI:** 10.3389/fsoc.2019.00019

**Published:** 2019-04-02

**Authors:** Gabriella Spinelli, Paul Weaver, Michael Marks, Christina Victor

**Affiliations:** ^1^Ageing Studies, Institute for the Environment, Health and Societies, Brunel University London, Uxbridge, United Kingdom; ^2^Groundswell Research Associates, London, United Kingdom

**Keywords:** aging-in-place, community, complementary economy, co-production, living lab, social innovation, time banking, community currency

## Abstract

Aging is continuously depicted as a force majeure event despite clear and robust premonitions of its coming. However, such depiction serves to justify the unpreparedness and inadequacy of policies manifesting in loneliness and isolation, unsatisfied demands in health and social care, lack of suitably inclusive residential and social facilities, and inequitable access to support and services. Recent years have seen an increase in social innovation that involves alternative transaction models, such as time-banks and circular economies. These initiatives represent collective responses to changes and challenges such as aging by identifying and innovatively capturing and exchanging locally- and freely- available assets with the intent to fulfill economic needs (more affordable goods and services), social ambitions (skills development and exchange, repurposing space, social inclusion, and cohesion) environmental aspirations (up-cycle) and psychological needs (sense of purpose, identity, belonging, recognition). Whilst it is often assumed that *ad hoc* measures are appropriate to resolve the challenges posed by an aging demographic, the learnt assumption that underpins this work is that aging is a systemic issue and ought to be understood, and resolved, in its context, not by producing niche- relevant policy and interventions, but considering the impacts it has on the whole society. Henceforth it is proposed that truly transformative social innovation for the aging population must consider and resolve the challenges of communities as these are where older adults can stay relevant socially and, in the presented approach, also economically. Through the review of four international case studies, a framework with four cornerstones has emerged. This includes the changing role of local and central governments, the models of value creation, co-creation mechanisms, and finally, technology, especially digital social currency. The concurrent presence of the four factors in the framework is not always a requirement for social innovation to emerge and flourish. However, the presented analysis suggests that all four themes have an impact even when not being direct agents of social innovation. The authors conclude by making a case for developing Living Labs for Aging-in-Place, to experiment and study proposed solutions for systemic challenges facing the aging population, grounded in community-led schemes.

## Introduction

Across post-industrial societies (and the political spectrum) population aging continues to be portrayed as a force majeure event despite clear and robust indicators that have long warned of its coming and inevitability. This portrayal serves to excuse the unpreparedness and inadequacy of policies, including lack of financial planning and investment, that manifest in insufficient residential and social facilities for older citizens, poor quality in health and social care, inequitable access to support and services, loneliness, isolation, limited choice, and unmet demands. Most seniors want to live as long as possible in comfort and safety in their own homes, but risk being institutionalized prematurely or having their cases medicalised for lack of social support, which adds to fear and suffering in old age and to pressures on hospitals through inappropriate admission and bed blocking (Age UK, 2016).

In the United Kingdom (UK), the landscape of social care for people in later life has changed rapidly following austerity-induced cuts to the social care budget. Not only has the budget been reduced in real terms, but it has also stretched to fulfill a growing demand encompassing complex co-morbidity cases that rise steeply with age. Statutory services are only capable of intervening in advanced cases where highly-qualified expertise is needed, usually when the client has become severely reliant and just before accessing residential care facilities. Usually, the client journey in the social care service is the expression of the individuals' wealth and social network. However, changes in family structure and relationships have contributed to a growing number of older adults without children. In total, it is predicted that there will be 2 m people aged 65+ in 2030 (Office of National Statistics, 2016a). We are, thus, at the cusp of a “crisis” in adult social care which, given these intersecting constraints and demands, will require not only novel solutions but novel ways of finding solutions.

Against this backdrop, the growing political (and wider) interest in the role of social innovation in addressing—and potentially averting—this looming crisis is explored. In particular, this work reviews and analyses a set of emerging social-economic experiments in developing inclusive and collaborative economies at local (community) scale, which focus on active and responsible citizenship and on developing sharing, caring, and circular (social) economies on principles of asset-based community development (ABCD) and reciprocity. Insights are drawn from four case studies of local initiatives that started as time banks, but which have developed in innovative ways, enabling them to thrive and address societal challenges of different type by attracting a wide set of actors and assets to their activities. Thereby, they have also avoided the disappointing but usual fate of most time banks, which often fail within 3 years of start-up. The four case studies examined here respond to diverse community aspirations and also differ in terms of longevity, penetration, and business models underpinning them. Through evaluation of the case studies, insights are provided on the future of complementary social (community) economies, their potential roles in new models of social care, and needed social and social policy innovation and experimentation.

This paper is structured as follows. The Materials and Methods section has three subsections. In the first of these (the landscape of social care today) today's social care models are reviewed and their design, operation and capacities to offer accessible, affordable and appropriate care and support options to all citizens as they age are evaluated. In the second subsection (social innovation) the growing policy interest in social innovation and in complementary community economy initiatives particularly as potentially offering new options to support aging-in-place is explored. In the third subsection (methodology) the approach of using case studies is explained and justified. The following section analyses the four case studies individually. In the discussion section, insights are drawn from a comparative analysis of the four cases leading to a discussion of emerging characteristics of an ecosystem for social innovation for aging-in-place.

## Materials and Methods

### The Landscape of Social Care Today

Social care can be broadly conceptualized as a portmanteau term that encompasses interventions that mitigate (or prevent) vulnerabilities in terms of compromised independence that are experienced by individuals rendered vulnerable because of age, disability, or disadvantage. In theory, social care interventions aim to both reduce negative outcomes such as vulnerability/dependence and promote positive outcomes such as enhanced well-being or quality of life. Any debate about the future or current state of social care needs to address three key elements: the characteristics of the individuals with support needs; the suite of services/interventions provided to address the identified needs (services in the home, community, or long-term care) and sources of funding/service delivery (public, private, and third sector).

Older people represent the primary group supported by the social care system because of difficulties with independent living as manifest by difficulties in undertaking basic activities of daily living such as dressing and toileting. Illustrative of the current situation are the statistics for England. In 2015/16 there were approximately 2 million requests to the relevant local authority for social care support of which 75% were from older people (Full Fact, 2017). Of the almost 1 million people receiving long-term support, approximately two-thirds are aged 65+. Approximately half of all social care expenditure is for those aged 65+ (Health Foundation, 2018). According to Kingston et al. (2018), the next two decades will present increasing demands on the social care system given the increase in the expansion of morbidity and predicted increase in the number of older adults living with multi-morbidity (four or more conditions). Kingston et al. (2018) offer a more nuanced view of future demands for care using a measure of dependency classified as high (requires 24 h care), medium (daily care) and low (less than daily care) along with independent. The next two decades will see the paradox of an increase in both numbers of individuals aged 65+ who are independent; those who are dependent and those with complex care needs consequent to increase in multi-morbidity noted earlier. This research reflects the potential increased need for social care resultant from the population aged 85 and older, which is estimated at 3.7% increase per annum (Wittemberg and Ho, 2015).

Both the formal social care system and care provided by families encompass the response to these care needs. Establishing robust data about trends in social care funding is problematic. However, it is suggested that expenditure in England fell by 6% between 2009/10 and 2015/16. It is unlikely that the next two decades will see increases in state funding to address these increases in demand. Family care-usually provided by a spouse or adult child-contributes significantly to the support of older people. Estimates of the financial value of informal care for older adults provided by family and friends are in the order of £57 billion in the UK in 2014, compared with approximately £23 billion from the formal carer sector (Office of National Statistics, 2016b). Projections of the future “supply” of informal carers suggest that it is unlikely to keep pace with demand; for example, it is projected that the care provided by adult children will go down while the role of spouse carers is likely to increase. Against a backdrop of increasing care demands, which are unlikely to be met by increases in funding or contributions from family carers, new models of care are required to ensure that vulnerable older adults can live independently and with dignity.

One potential component of a robust model of social care is to look at vulnerabilities that compromise independence or quality of life across the life course rather than focussing upon specific age groups. We argue that one deficiency in our current models of social care and the ensuing debates about funding is that it locates vulnerability to specific age groups, thereby setting up competition or conflicts between generations rather than taking a life course approach. One illustration of how vulnerability to compromised quality of life is not the sole prerogative of a specific age group is that of loneliness. Recent data from the Office of National Statistics indicates that loneliness is not merely a “problem” of old age but is experienced by adults of all ages with young adults recording the highest prevalence (Office of National Statistics, 2018). Interventions need to focus not just on older adults but on all age groups and to operate at macro community levels as well as on individual risk factors (Gov.UK, 2018; Victor et al., 2018). Identifying solutions with the potential for scaling up or out is also a topic of discussion and strategic developmental by the European Innovation Partnership on Active and Healthy Aging which has established six actions groups to define the challenges and organize collaborative work (European Commission, 2015).

### Social Innovation

Social innovation is a process that entails ‘doing things differently’ and involves change in social relations and systems (Haxeltine et al., 2015). By extension, initiatives that include new social relations for ‘doing things differently’ can be considered as ‘socially innovative’ and the new ways of thinking, organizing, acting and interacting they introduce can be considered as ‘social innovations’. Social innovation as a process responds to at least three different stimuli: intrinsic needs people have (e.g., for self-determination, social bonds, and to be engaged in activities and relationships they find meaningful); perceived failings of mainstream systems or gaps in mainstream provision; and, change in the wider socio-material context that present problems or opportunities (Weaver et al., 2017). Kemp et al. (2016) argue that social innovation is often a reaction to institutions and developments that are perceived as “dehumanizing.” People who form social innovation initiatives are often drawn together by shared values, shared ways of seeing, framing or reframing challenges and shared visions of how to address challenges ‘differently’. Through their initiatives, social innovators seek to demonstrate and diffuse new forms of social relations (or to revive older forms of social relations that are no longer widely practized) and new ‘proto’ institutions that might challenge existing institutions, altering, replacing, or offering alternatives to these (Haxeltine et al., 2017) or providing complements that make existing social systems work better (Weaver et al., 2017). Recent progress has been made in conceptually defining social innovation, distinguishing types of innovation by the scale and scope of change sought and the extent to which the innovation is complementary or radical to current social institutions (Marques et al., 2018). Weaver and Marks (2017) note that the effectiveness of social innovation as a counter-force depends on whether initiatives that propose and demonstrate alternative social relations, social models, and systems are able to sustain and scale and, in the process, can still retain their humanizing qualities. Key dimensions of social innovation, in this respect, are the scope, scale and intensity of activities and the durability or longevity of initiatives. These relate to the potential of social innovation to contribute to transformative change at societal- or systems- levels. Scaling of social innovation can occur through processes of scaling up and scaling out. For social innovation initiatives whose proponents hold transformative ambition, ways must be found to sustain their initiatives and, ultimately, to scale them up and/or replicate them.

Some social innovators aspire to strengthen community resilience and to meet individual and community needs by organizing networks of sharing and reciprocity, where individual and organizational members exchange services and skills (e.g., time banks) and make use of each other's additional facilities and equipment, such as meeting rooms, tools, and internet access. Recent years have seen an increase in social innovation that involves transactions that reduce energy and resource waste (e.g., circular economies) and peer-to-peer transaction models that involve collaborative sharing of goods and services (e.g., sharing economies) and less reliance on fiat money as a medium of exchange (e.g., solidarity economies). Such initiatives represent collective responses to change by identifying and innovatively capturing and exchanging locally- and freely- available assets with the intent to fulfill economic needs (more affordable goods and services), social ambitions (skills development and exchange, repurposing space, social inclusion, and cohesion) environmental aspirations (up-cycle), and psychological needs (sense of purpose, identity, belonging, recognition).

Time banks and related initiatives developed therefrom, which are highlighted in the case studies that inform this research paper, are networks of individuals and organizations, usually within a local community, whose members exchange services. They are outcomes of social innovation processes and represent proto type institutions involving forms of social relations built around principles of reciprocity. In time banking, time is used as a community currency and unit of account and all exchanges are considered equal in value irrespective of the skill involved or level of complexity (Lasker et al., 2011). Time banking also recognizes that capabilities and the amount of time and energy an individual can devote to making contributions vary across an individual's life course and this applies also to needs. This gives scope for both horizontal service exchange, where members exchange services in the current period intra-generationally, and vertical or inter-generational service exchanges that involve the ‘banking’ of earned hours for redemption later. These aspects have led to claims that time banking could help strengthen bonds within communities and build community resilience (Cahn, 2004; Shih et al., 2015), could be deployed to support social and economic inclusion for individuals otherwise marginalized or excluded (see Drakeford and Gregory, 2010; Marks, 2012; Weaver et al., 2015; Skropke, 2016) and could be useful in building missing support systems for social welfare and health care systems.

Social innovation initiatives, such as time banks, are attracting increasing policy attention because they are seen to hold the potential to address a growing range of societal challenges that are difficult to address through mainstream approaches. Many of today's pressing societal challenges have systemic pathologies linked to prevailing development models. This makes them less tractable to solutions developed from mainstream logic. Social innovation initiatives are seen as a prospective source of novel solutions because solutions are designed from values and principles different from those of mainstream institutions and systems. Furthermore, the resourcing needs of social innovation activities differ significantly from those of more mainstream activities. Social innovation initiatives typically require relatively little money, instead using otherwise wasted or underutilized resources already available within communities as inputs to their operations, such as labor, facilities and equipment of their members, which members are willing to share and exchange (Weaver et al., 2017). These kinds of initiative—based on mutual aid, reciprocity, and asset-based community development—also resonate strongly with concepts of care-in-community and aging-in-place. This attracts policy and public interest, especially in times of austerity and increasing demand on mainstream services, when public finances and services are stretched.

Policy interest notwithstanding, harnessing social innovation organizations and initiatives is far from unproblematic. Part of the challenge lies with establishment actors, who sometimes seek to co-opt initiatives to serve specific departmental or agency agendas not fully appreciating that by jeopardizing the independence and autonomy of initiatives they risk destroying the basis of grassroots support which is the source of both the useful and innovative potential they seek to capture. Attempts to co-opt by individual agencies also ignores that many social innovation organizations and initiatives have a cross-cutting scope. Failing to provide cross-agency financial support (which would make this an easier lift for each agency) and to guarantee long-term support (difficult when policies and governing parties can change) risks narrowing the scope of social innovation organizations and damaging their prospects to contribute to more wide-ranging and longer-term societal and systems changes.

Another part of the challenge lies with social innovation organizations and, more specifically, with the relationships on the one hand between different social innovation organizations and, on the other hand, between local manifestations of particular types of organization and the membership organizations that often emerge to represent them. Social innovation organizations often formalize around an “approach” or “tool” they develop and/or a domain of action where they apply their approach. In seeking to promote their specific approach, they often over-claim what they achieve. They can also become protective about “their” approach and “domain” and often end up competing with other initiatives when collaboration would deliver better outcomes more cost-effectively. The tool and the identity of the initiative can become intertwined—as is the case with time banking—and these can become separated from the actual mission. The purpose can mutate from delivering positive social impact and innovating continuously to this end, to one of promoting the tool *per se*.

When membership organizations emerge to represent local initiatives, these problems can deepen. The interests of the paid professionals—who seek to develop income streams to support their operations—do not necessarily align with those of the local initiatives that, ostensibly, they represent, and should support. The actual role played by membership organizations can become parasitical and perverse, since their income stream depends on creating dependencies. In the case of time banking, control is exerted on local manifestations by creating dependence on software supplied via the membership organizations and by requirements to adhere to rigid models of time banking approved by the center. The membership organizations with individual access to activity data across all local manifestations have most to lose if the data do not support their claimed levels of membership and social impact. In the case of time banking, there is no credible evidence made available by the national UK membership organization, Timebanking UK (TBUK), to back its claims that time banking is a growing movement that delivers wanted social impacts cost-effectively.

Vested interest on the part of the membership organization precludes using the data as a resource to support time banking experiments that could improve performance. This frustrates innovation and deters sponsors. It also leads to a misleading narrative on the part of the membership organization that it only requires an initial investment to establish a local time bank and, once established, this will add permanently to community infrastructure. In reality, virtually all time banks fail in their first 3 years. There have been more than 500 times bank deaths in the UK since time banking was introduced around 20 years ago. TBUK claims to represent around 300 time banks currently. The actual number is estimated by investigative researchers to be around half this number with fewer than half of these evidencing even modest levels of activity per member.

### Methodology

The methodological approach involves case studies of four social innovation initiatives that started as time banks but have evolved into more broadly-based community economy initiatives. The case studies are framed as “ongoing experiments” in developing community economies and as success cases since, unlike most time banks that struggle to survive even for 3 years, these have or are developing business models that have enabled them to sustain and expand the scope and impact of their activities. These are interesting because they have continued to innovate and evolve in the processes of seeking to leverage their positive social impact and to secure their financial sustainability.

If an initiative can grow and attract more citizens and organizations to join, it is more likely to be able to offer a wider set of asset-sharing opportunities to participants, which will help it sustain, attract additional participants and grow further. Conversely, if the rate and level of growth of the initiative are low, the sets of participants, assets and opportunities may be too limited to attract others to join, and the initiative is unlikely to survive. Other factors known to be important for sustainability are the level and trajectory of transaction costs (e.g., high costs for few transactions and growing marginal transaction costs) and the limited capacity of the initiatives to demonstrate actual activity levels and social outcomes accomplished. Against this backdrop, our objective is to establish framing conditions and principles for designing and implementing community economies that can address these challenges.

To accomplish this goal, this research sets to learn from initiatives that have sustained and grown over time. A multiple case study comparative approach (Yin, 2003) was adopted selecting “success-cases” (Stake, 2000; Brinkerhoff, 2003; Murphy, 2016) to understand what these long-surviving initiatives do that is different and that contributes to their sustainability. The four community economy initiatives that form the basis of the comparative case studies are: Lewisham Local (LL), Give&TakeCare (G&TC), the Hull and East Riding Mutual Aid Network (HERMAN) and Partners-in-Care (PIC). All are UK based with the exception of Partners-in-Care which is based in the United States. The case studies were identified through a process that involved screening currently-active time banks in order to find those that have sustained beyond 3 years, are very active and are innovative in developing wider community economies (HERMAN, LL) and/or in addressing aging in the community (LL, G&TC, PIC). These initiatives are framed as “ongoing experiments” in developing community economies, but also as success cases, since, unlike most time banks that struggle to survive even for 3 years, these initiatives have developed in innovative ways, enabling them to thrive and address societal challenges by attracting a broad and diverse set of actors and assets to their activities. In justifying the selection of the case studies and their attribution here as social innovation cases reference is made to the two distinctive factors identified by Marques et al. (2018), “inclusiveness” and response to “need”. Individuals often marginalized are participants in the four case study schemes (e.g., older adults and those with physical and/or mental health issues who, otherwise, are vulnerable to social and/or economic exclusion) and the schemes are driven by community needs, including the needs to support aging-in-place and to relieve loneliness, poverty and other forms of deprivation.

Identifying and framing these as “success cases” enables to draw on methods of implementation science to identify factors relevant as drivers, enablers, barriers, and success that influence the implementation process (Nilsen, 2015). Timelines, critical moments and turning points in the evolution of initiatives are also reviewed as well as the dynamic interplay between the initiative and its wider socio-material context. Table 1 summarizes the four selected cases in relation to basic attributes. A detailed narrative description and analysis of each case is provided in the next section. Through their subsequent comparison, as ‘best practice’ cases, this inquiry seeks to develop insights about framing conditions and design principles for complementary community economies that could be used as testable hypotheses in next-stage formal experiments organized along the lines of Living Labs (Almirall and Wareham, 2011).

**Table 1:** Characteristics of the 4 case studies.

	**LL**	**G&TC**	**HERMAN**	**PIC**
Rooted in time banking	✓	✓	✓	✓
Rural (R), Urban (U); Contiguous Site (CS), Multi-site (MS)	U, CS	U, MS	U, R, CS	U, R, MS
Start year	2015	2015	2012	1993
Targeted demographic group	×	✓	×	✓
Proprietary technology (PT) or off-the-shelf technology (OTST)	OTST	PT	PT	PT
Formal participation of local authority	✓	×	×	×
Immediate reward (IR) or delayed reward (DR)	IR	DR (plans for IR)	IR	IR
Inclusive relationships	✓	✓	✓	✓
Businesses involved	✓	×	✓	✓

## Case Studies Analysis

This section presents a description and analysis of the four selected cases. The cases have been chosen as they all have historical roots in the time banking movement, recognizing the importance of attributing value to underutilized resources such as time. In time bank terminology, members earn hours benefitting the host organization in some capacity and in return, receive benefits in services provided by other time bank members or from the time bank itself. Another common feature of the four innovation cases is that they are all place-based and the identity of the community is strongly informing the type of initiatives and projects occurring in each scheme.

### Lewisham Local

Lewisham, with a population of more than 300,000 people is located in South-East London. Lewisham Local (LL) is a scheme addressed to local needs and aspirations. As Lewisham has no borough-wide local trusts to invest money into meeting local needs and as local voluntary and community organizations have faced austerity-induced reductions in grant funding from central and local government, the need has been felt in Lewisham to encourage place-based giving on the part of local individuals, businesses and enterprises to support community organizations and their initiatives. LL is a place-based scheme that was launched in 2009 on an inclusive definition of assets and capacities to contribute to Lewisham as a place and as a community. The basic principle is that all citizens of Lewisham, whether individuals or organizations, have something they can contribute either in cash or in kind, such as time, skills, tools, materials, facilities or spare capacities.

Since its inception, Lewisham Local has been led and developed by a cross-sector collaboration of Lewisham based organizations, including, Goldsmiths University of London, Rushey Green Time Bank (a 20 years old Lewisham based timebank), Voluntary Action Lewisham, Lewisham Education Arts Network, and South East London Chamber of Commerce. Lewisham Council is also a formal partner in the initiative with this being a unique feature in comparison to the three other cases reviewed in this paper. The collaborative meets every 2 months to plan the strategy for the scheme.

The collaborative was initially sponsored with seed funds by City Bridge Trust with the purpose to trial the development of a local giving scheme in Lewisham. Management support, governance, IT, and premises are donated in-kind by members of the collaborative steering group. Most of such assets are donated by Rushey Green Time Bank, which hosts Lewisham Local (LL). LL employs one part-time Development Lead, who reports to the Collaborative. Day-to-day management of the scheme is undertaken by the Rushey Green Time Bank. Having established a long-standing reputation among residents and been trusted by the Local Authority, Rushey Green Time Bank was a good strategic partner for Lewisham Local with whom it shares assets to address local needs. Rushey Green Time Bank is also well known in the local voluntary and community sector and has adapted to be an agile and forward-thinking organization.

So far, the scheme has had a diverse target audience, and the only common denominator among participants has been the geography of Lewisham; i.e., people or organizations living, working, studying or conducting operations in Lewisham. Keeping a wide definition of contribution has fostered a collective commitment to getting involved and strengthened the community. Currently, LL has a network of 300 businesses that offer discounts to 5,000 individuals who volunteer for local charities. The LL card is the means to recognize and reward acts of community participation and caring. Local businesses offer discounts on goods and services to holders of the LL card. In return by buying locally, the LL card increases local business activity. In addition, Lewisham community members consider the businesses are participating in the Lewisham local card as partners. A network of 180 charities and non-for-profit organizations sponsor initiatives that support vulnerable groups and organize environmental and social actions. One such example is the campaign to reduce single-use plastic waste. This issue grew in public consciousness following the increased publicity from the broadcasting of Blue Planet II toward the end of 2017. Lewisham Local responded quickly and called upon the network of community-minded businesses to develop free-water refill stations to help reduce single-use plastics.

LL has begun to collect data on the type of needs and interventions organized and delivered to better evaluate the scheme and efficiently identify the direct use of funds secured and needed.

Local Lewisham fosters creative connections through multi-stakeholders' participation and follows a simple rule of relevance which is that initiated projects ought to be place-based. Through time LL has considerably grown its local business network, and plans include strengthening the relationship with larger local employers.

Future priorities of Lewisham Local include:

– Developing a financial giving scheme to provide funds for local organizations who help to address issues such as poverty, especially poverty among ethnic minorities in Lewisham.
– Improving internal processes to gather data about local giving from individuals and businesses and how projects impact on community.
– Cultivate leadership by expanding the skills of community members and broadening local leadership.
– Procuring and deploying a technological platform able to reduce administrative and support service and asset exchanges seamlessly. Lewisham Local is the only case study site without a proprietary technology to support activities. Addressing this limitation will provide data to help leaders to understand what works best to achieve outcomes and why specific actions are effective.

### Give&TakeCare CIC

Give&TakeCare Community Interest Company is a registered Company incorporated in the UK in 2015. The Give&TakeCare (G&TC) scheme arose as a disruptive solution to the care crisis in the UK. The aims that motivate G&TC are:

– Enable older adults to manage long-term conditions away from residential facilities and hospitalization for as long as possible;
– Motivate, educate, and empower citizens to “contribute” to their care and that of others in their communities given the unprecedented demographic changes;
– Recognize the key role of the voluntary sector in supporting older adults and carers and create an additional income for them given the reduction in legacy and local authorities' grants.
– Acknowledge and support informal carers who even if unskilled and unsupported still provide £132 billion worth of care for the year 2015.
– Provide a solution to the needs of those aging without children or immediate family

In February 2016, the company won a major UK funding competition launched by Innovate UK under the “Long term social care revolution”. The contract allowed G&TC to be operational for 31 months until successful completion in August 2018. By then G&TC had established four sites either directly, or in collaboration with other social enterprise and charities in England, it had organized over 1500 hours of care and support for older adults and had involved around 500 partners as caregivers and care recipients. G&TC has secured some continuation funds from the BetterCare fund in the Royal Borough of Windsor and Maidenhead, the Design Council and Unltd and a philanthropic grant. In addition, an interim agreement with Vista ltd in Leicester has allowed continuing to operate the Leicester site.

To achieve its aims G&TC offers a time banking scheme, allowing those who take part to create a Care Savings Account, accruing “care-credits” for their future. This feature, unique to G&TC among the four case study sites, can be considered akin to a pension; volunteers give up their time now and make provision for their future care. The incentive of the Care Savings Account is a vehicle to encourage people to care for each other now and create future resources for their own care outside of public funds. However, the delayed reward has been unsuccessful in attracting new individuals who were new to volunteering. The scheme offers more person-centered care for the older adults as care receivers and care givers are matched by their needs and skills and the support provided is in response to the care receivers' request. The scheme is also available to family carers who carry out the largest proportion of care in the UK.

A significant challenge was getting organizations to sign up to the scheme. This was particularly true of large national charities who were deemed vital in achieving the scale needed to produce a sustainable scheme. It was found that trustees/directors are traditionally suspicious of new schemes that can only provide short-term financial support as was the case with the G&TC subsidy. The national organizations approached by G&TC were also keen to avoid extra costs of administration, they were risk-averse and quite conservative in the model of income generation they implemented and wished to consider. In addition, many national charities have introduced paid services (gardening, befriending etc.) at varying costs (£15–20 per hour) and the implementation of a free befriending/domestic support service such as G&TC, represented a threat to their current modus operandi, despite their volume of income through paid services being quite small. Owing to the delays caused by trying to persuade national charities to engage with the scheme, G&TC decided to work in collaboration with local organizations rather than operate at a national level. This choice has inevitably impacted on overall scheme sustainability, which represents a differentiating point that sets G&TC apart from other time banks. While other time banks rely mainly on an IT platform to match people and on grants to support the operations, G&TC initially set out to be financially independent using a small administrative contribution from all care recipients in the scheme. The contributions amount to an annual membership fee of £5 plus £1 for each hour of care received. The total cash contributions are intended to support G&TC and the associated local organizations. The main purpose of the financial contribution was to cover the salary of a community coordinator, a key role given the potential vulnerability of older adults and the safeguarding needs this implies. Wide consultation with service users, their families, and charities were initiated by G&TC to gather feedback and informed the scheme and the services provided. The difference between traditional befriending services and G&TC were highlighted by the stakeholders and what seems to make G&TC distinct were:

– G&TC aims to be client-centered and identify a volunteer or a team that can support each client. Volunteers and care receivers are encouraged to exchange contact details as they are members of the same community and friendships have a great value for older adults who feel lonely.
– The befriending services offered by G&TC span a wider range including basic practical help, supporting older adults in daily activities they are no longer fully able to undertake (e.g., administrative help, walking pets, tidying up, car lifts).
– The intensity of the exchange between clients and volunteers is completely determined by the clients' needs, making the scheme flexible.

G&TC developed a bespoke IT platform which is used for matching care receivers and care givers and tracking care hours exchanged.

The potential scalability of G&TC depended on engagement and collaboration with associated organizations to offer the scheme to their existing members and to recruit additional members with the incentive of becoming partners to build a Care Savings Account. After the take up by charities and/or not-for-profit organizations (NFP's), the sustainability of the scheme depended on the hours of care exchanged in the system, which in turn is the measure of the adoption by end-users (givers and receivers). The uptake of G&TC is also an indicator of significant social attitudinal and behavioral change toward community self-reliance. Equally important is the ability to transform older adults from traditional care recipients to committed and socially engaged care givers, in whatever capacity possible.

### Hull and East Riding Mutual Aid Network

The origins of the Hull and East Riding Mutual Aid Network (HERMAN) lie in the Time Bank Hull and East Riding (TBHER). TBHER was established with small-scale local funding in 2012 originally to provide mental health support services, but its scope broadened and, instead of focusing on one target group, took a whole community development approach with the strategic goal to reconnect and rebuild the whole community in Hull and East Riding using a mutual aid network (MAN) approach based on network members sharing resources and assets. The role of TBHER also broadened from a time bank to connecting different organizations locally to create synergies and maximize positive social impacts through asset sharing and joint working. There are mutual lines of influence here between TBHER and the International Mutual Aid Network Programme, which seeks to develop pilots in towns and cities around the world. Through TBHER and its efforts to develop HERMAN, Hull is the only European MAN pilot site within this network and one of only eight pilot sites globally.

TBHER has partnered with the University of Hull and the Webb Memorial Trust in an initiative that engages local people and organizations to help shape a shared and inclusive vision. The “#thehullwewant” initiative offers opportunities to voice aims, aspirations and concerns, but is also used to help change dominant perceptions, cultures and narratives from those of “scarcity” and “deficit” to “abundance” and from “passivity” to “activity” in realizing the hopes and vision for Hull. Part of the cultural change is to support a shift in thinking about how needs can be satisfied and how opportunities can be created. MANs and Asset-Based Community Development (ABCD) approaches stand the usual solution pyramid on its head so that sharing sufficient assets is the first option and money is the last option when finding a way to address a need or solve a problem. Components of HERMAN include exchange mechanisms and platforms for sharing, borrowing/lending, swapping, time exchange, and skills exchange. These use currencies other than regular (fiat) money as units of account, means of exchange and facilities for credit and saving, such as time and local currencies.

TBHER/HERMAN is currently in receipt of a three-year Big Lottery grant (ca £70 K annually). The initiatives can draw, also, on the time, talents, and assets of their members. Working with established local organizations and being recognized as an important partner lends legitimacy and credibility to TBHER and HERMAN. TBHER no longer uses time banking software supplied through the national membership organization for UK time banks, TBUK, but has its own provider. It also makes use of open-access software and platforms to log members and assets. These arrangements have become necessary to provide TBHER with autonomy and agility of action. A research partner supports fund-raising, monitoring, evaluation, and reporting activities on a largely pro bono basis.

The main beneficiaries are the individual and organizational members of HERMAN and those who are served by HERMAN's members, such as the tenants of housing associations benefitting from exchanges and befriending services. TBHER/HERMAN reaches out especially to neighborhoods and areas that score highly on indicators of multi-deprivation and experience community tensions on age, ethnic and/or religious lines, and by placing “ambassadors” to work in neighborhoods and build links with local organizations, programs and projects. Ambassadors link up with the social prescribing networks so that primary health/care professionals can refer clients. The same applies to the local job center. TBHER/HERMAN has reached out to help integrate migrants and newcomers to Hull into the host community and to offer social and economic inclusion. An example of the process leading to identifying beneficiaries is the befriending scheme that TBHER/HERMAN has initiated with a local organizational member, Pickering-Ferens Homes (PFH), which provides housing for older people. This involved reaching out through the communications channels of PFH, TBHER/HERMAN, the local Older People's Partnership (a partnership of organizations and groups concerned for welfare of seniors) and health groups, such as the Freedom Stroke Group (comprising victims of strokes and their carers) to identify potential beneficiaries and to build a befriending network to combat loneliness and isolation. TBHER and its initiatives, such as HERMAN, are governed by a Board of Trustees.

TBHER was a recipient of a local authority grant in its establishment phase and formed a part of the community infrastructure for a secondary collaborative economy as part of the local anti-poverty strategy. TBHER is well-known to community and anchor organizations locally and is building a reputation as an umbrella organization with capacities for orchestrating innovative responses to local needs involving different organizations and approaches.

As a pilot MAN and the only such pilot in Europe, HERMAN is the most significant innovation of TBHER and is a work in progress that engages continuous innovation and learning. HERMAN differs from other ABCD initiatives because it seeks to attract a diverse range of individuals and organizations to participate in the local collaborative economy it seeks to create and capitalizes on the diversity and complementarity among their assets and needs. The scope for creativity lies in addressing different challenges together rather than in silos. It also proposes to use advances in ICT as enabling technology to organize exchanges and lower transaction and safeguarding costs.

The main factor in continuity is that TBHER/HERMAN sees social innovation as a process that addresses ever-changing challenges in an evolving context. It seeks to support the emergence of partnerships among stakeholders in relation to different challenges that the community prioritizes and relationships among these rather than promoting a single tool or approach or addressing a single target group or domain of need.

TBHER has learnt that time banking as a stand-alone approach is too limited and too static and that there cannot be universal solutions to ever-evolving challenges, especially when these are multi-faceted and many of these facets are specific to local conditions and contexts. The need instead is to build capacities and processes within the community to identify priority challenges and enable these to be addressed by stakeholders with local knowledge using local resources. The next steps will be to introduce digital currency to the mix of community currencies, test different models for managing the currency to maximize its potential to leverage engagement and positive social impact, and test models for governing and valorizing the data generated.

### Partners in Care

Partners in Care (PIC) is a time exchange community that offers services to Maryland (US) older adults and individuals with disabilities. It is the longest running operation of the four case study sites, having begun in 1993. It has grown to a membership of 3200 people that includes PIC staff, seniors of all ages, their family members and friends, and other community members contributing to the time exchange and the organization. PIC has a number of special programs including “Repairs with Care,” which provides handyman support; “Ride Partners,” which provides transportation to older adults with members using their own cars and “Member Care,” which provides personalized support such as home visits, help with paperwork, light housekeeping, pet care, grocery shopping, and small social gatherings. Service exchanges and specialized programs improve the care of current older adults. Providers of service earn hours that later can be used for their care in the future, supplementing the nation's social security payments provided to seniors. PIC provides opportunities for everyone to benefit and contribute to older adults' care regardless of income or job status. Membership in PIC is voluntary and not formally linked to national social security systems (see www.partnersincare.org; Weaver et al., in press).

The evolution of PIC as an organization involved many ups and downs with resilience and grit characterizing the three founders and its dedicated time bankers. Early on it was named the Service Credit Banking program (for Seniors) and came under the auspices of a local hospital where it was located. This provided instant visibility and credibility for the fledgling organization, enabling it to attract small grants and donations as well as in-kind services such as marketing and promotion. Early time bankers signed on to offer rides with no promise to be reimbursed for expenses such as petrol costs. After 4 years, the partnership with the hospital ended with only 30 days-notice. PIC members responded to the challenge: a new location was identified and funding was secured to maintain operations. Nonetheless, this experience was profound. In order to maintain levels of self-sufficiency and sustainability, PIC set forth an operating principle to seek out many and various small grant awards to fill service gaps identified by older adults and fellow time bankers with no expectations of sponsors to carry the full burden of project funding or to fund projects indefinitely. In other words, PIC is run with older adults as labor and reciprocal transactions. It decided not to become dependent on grants or major support from any one funder. Further, PIC has a policy of maintaining the share of grant funding (philanthropic and statutory) in the overall mix of organizational income at or below 40 per cent. This principle has served PIC well over its 25 years of operation (Hogan, 2017).

PIC is currently supported by a combination of government and foundation grants. In addition, PIC has developed partnerships with other NGOs to provide valuable services to older adults. Over time, for example, PIC developed a solid relationship with the Anne Arundel Volunteer Center (AAVC). AAVC pairs volunteers with local NGOs, so their respective organizational missions complemented each other. PIC became a major source of AAVC referrals. This kind of mutual working relationship extended to dealings with statutory bodies.

In addition, PIC is able to maintain financial sustainability in part because it established a social enterprise that uses the sweat equity of its time banks members. The PIC boutique, is staffed primarily by time bank members who earn hours supporting the business. The boutique provides income close to US $500K annually (about 35% of total agency revenue) in support of PIC programming for older adults in the community. The time bank owns the profit-making boutique. PIC trains all staff who work in and who support the boutique.

PIC is faced with new challenges as the organization continues to evolve. There is growing recognition that as demands for services grow, new and different recruitment efforts need to occur. PIC is exploring expanding its time bank base to include younger adult millennials and active older adults. The need for a greater evidence-base of the impacts of its model is increasing as PIC seeks partnerships with the local and regional hospital-based systems interested in the PIC model to expedite hospital discharges and prevent unnecessary emergency room or other acute care needs. These kinds of partnerships require a greater focus on outcomes and perhaps recruiting a new kind of volunteer who commits to a set schedule of tasks to be performed as and when these are needed.

## Discussion: The Emerging Characteristics of An Ecosystem for Social Innovation for Aging-in-Place

**Figure 1:**
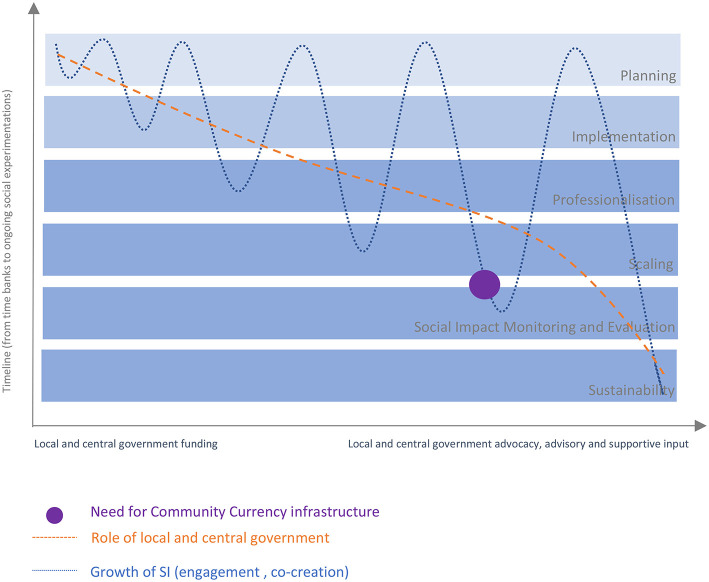
An integrative framework for the development of Social Innovation.

In 2007 colleagues at Philips Research Europe in Eindhoven who initiated the carelab, a residential assistive module to monitor and support older adults, stated that, given the exceedingly fast growth of the 60+ demographic segment, there would always be a lack of carers. This was the premise justifying their investment in technology to support later life (de Ruyter and Pelgrim, 2007). While it is only sensible for societies to consider how the deployment of technology may support aging, starting from making technology more inclusive, the fulfillment of some human needs by technology is hardly imaginable, such as the need for personal identity or to feel relevant and valued as a member of society. In the exploration and discovery of how to deal with such unprecedented change as that represented by population aging, social innovation and ecosystems capable of fostering social innovation might make pivotal contributions. In the following discussion, four cornerstones of an ecosystem for social innovation for aging-in-place are identified. These are drawn from similarities and differences highlighted by the case studies and are discussed as factors relevant to the relative success of the cases as social innovation initiatives that are contributing to strengthening communities and social systems. In order to simplify the discussion, the four cornerstones are considered in artificial isolation as constituent elements of what, actually, are complex and interconnected ecosystems. A cautious approach is adopted in describing the cases as ‘relatively successful’ because, even if all of the studied schemes have sustained their operations for longer than most time banks, in absolute terms the operating periods involved are from 4 to 25 years. Three of the four organizations considered received seed funds from local bodies. G&TC received funds from central (national) sources, which came with a mandate to initiate nationwide operations. However, G&TC organizers soon realized that a place-based strategy with almost self-selected communities was the only viable option. The four cases help illustrate how a systemic solution to community cohesion may as well be a sustainable strategy to the aging tsunami and the considerations presented in this section start to sketch “communities” as self-organizing and self-caring organisms. Figure 1 visualizes the relationships between the four components considered essential in this paper for the creation over time of ongoing experiments in care-in-community and care-by-community. The axes represent the timeline of social innovation and the role of central and local government over time. The blue curve traces the extent of co-creation and co-delivery, set to grow over time. While recognizing that professionalization (e.g., the intermediary role) is present from an early stage in some cases, it has been learnt that monitoring activities and evaluating social impacts are complex administrative tasks that a digital currency infrastructure can help to support and simplify. In Figure 1 the introduction of digital infrastructure coincides with when the level and complexity of activities make it no longer practical for monitoring, coordination and evaluation to be handled manually. Figure 1 also visualizes the dual and shifting role that local and central government can play initially as investors in establishing schemes and infrastructures and subsequently in supporting sustainability and scaling of the social innovation, including by providing continuing income as service commissioners.

### Model of Value Creation

The inadequacy of the linear model of innovation has emerged under the added pressure of unprecedented global competition and compelling societal and environmental changes (Freire and Sangiorgi, 2010). In the last 20 years the single value creation model has been challenged and has evolved to encompass open innovation models that feature *Virtuous Ecosystem Participation* (Herskovits et al., 2013, p. 636); an innovative network of partnerships aims to create value (e.g., innovation) even through collaborations with organizations traditionally perceived as competitors. Organizations have sought Virtuous Ecosystem Participation to include talents not strictly related to their core area of business and in so doing achieved multiple streams of value creation and organizational ambidexterity, for example, the capacity to be agile and respond with a continuous programme of innovation to the changing surrounding (Ortt and van der Duin, 2008).

Albeit with some delays, social innovators have also embraced the changes just described but under different contextual pressures than that of businesses, for example, the frustration created by diminished public budgets, the growing isolations of certain social groups, the decline in civic participation and the void determined by underdeveloped public policy. The four case studies described, starting from time banking, have applied a non-linear model of value creation that recognize undervalued assets. By doing so, these assets have been legitimized, empowering community members who are cash poor or unable to contribute through financial means to access other means of participation.

The Value Constellation Model (Normann and Ramirez, 1993) has been held as a possible blueprint for a sustainable framework able to foster and maintain environments of multiple non-linear relationships. A key requirement of such a model is that partnerships in the forms of co-design, co-production and co-creation underpin the sustainability of the proposition (Cottan and Leadbeater, 2004; Murray et al., 2006).

Although at a different stage of the maturity cycle, all four reviewed case studies have reduced the gap between innovation and implementation by adopting a co-creation and co-delivery model that has enabled the stakeholders to design, deliver, and reflect iteratively on each strand of work, with the ability to calibrate, reorient and dissolve activities no longer fit for purpose. This agility resembles that associated with “Living Labs,” approaches used in contexts that are complex, evolving, uncertain (and that, therefore, entail risk that novel solutions will fail), which involve stakeholders from the start and throughout the innovation cycle and that experiment with solutions in context and in real time, learning about these and refining promising approaches (Almirall and Wareham, 2008; Galli, 2010). Living labs lend themselves to be a useful contemporary methodology in design for social change as they frame the intense relational work undertaken by emerging social enterprises needed to support aging-in-place, empower local intermediaries such as the social actors who are described in the analyzed cases, and allow for data collection and iterative design interventions in real time. The combination of Living Lab methodology and non-linear models of value creation may represent the theoretical foundation for the development of social innovation models for aging as they cope with the uncertainty of changing local and community contexts and with the need to identify alternative and supplemental value-adding assets held already within local communities that can be mobilized, developed and deployed to address local challenges (e.g., the spare capacities of local people and organizations). The diversity of value-adding resources has been acknowledged in HERMAN as well as in LL where focusing on straightforward reciprocal exchanges was replaced by a more complex and inclusive groups of active citizens, organizations and their assets, exploiting the notion that diverse talents and resources can all contribute. Conversely, PIC and G&TC followed a more narrowed-down initial approach to exchanges, for example to focus on the targeted aging population and its direct care givers.

### Co-creation

Participatory processes of asset creation are another major cross-cutting theme that guides the development of social economies. Theories of co-production inform time banking and other social economies. Co-production is an asset-based approach that rewards contributions and alters the notion of work within human service programs and communities (Cahn, 2004). Its primary aim within social services programs is to enhance service participant engagement, to sustain the engagement while enrolled and to prepare participants to succeed post-discharge. By intentionally involving participants in activities where they can contribute and use their assets, and by encouraging, recognizing and rewarding their accomplishments, participants gain new life and work skills (Marks, 2012) and move from being passive recipients of externally-provided welfare benefits to becoming active and productive participants in locally-generated welfare creation (Weaver and MacDonald, 2018). There is some limited evidence in the health care area suggesting improvements to patient outcomes when patients, including older adults, partner with medical professionals in their own health care (Kyriacou and Blech, 2003; Lasker et al., 2006). Simon and Boyle (2008) have argued that co-production offers an approach to address the emerging crises in health care and adult social care. An intervention framework has been developed, which includes practices and strategies to help guide practitioners on methods of empowering service participants (Marks, 2012).

The potential impact of co-production strategies on organizational and community development is less conceptualized and understood. Time exchanges that tap the unused labor of community members to support and grow organizations (e.g., citizen-organizational co-production) and improve communities (e.g., citizen-community co-production) are recognized as important venues for contributions (Marks, 2012). Time banking principles (e.g., people as assets, reciprocity, moneyless exchange) are also identified as holding a potential to further ABCD by mobilizing communities' own assets and resources (McKnight and Kretzmann, 1996). This holds true whether these principles are operationalized through time banking or, given that time banking on a purist model is difficult to sustain because of high transaction costs, these are taken up as elements of novel solutions that only reflect this time banking heritage, such as through broad-scope complementary community economies enabled by digital community currencies.

Case examples studied in this paper begin to shed light on processes and tools that could extend the reach of co-production to develop and improve communities and their collective response to critical challenges. Highlighted projects were often faced with few or no resources to invest in addressing local needs (e.g., LL and PIC). This created the need to attract local giving in the form of small grants, in-kind contributions, and donations of time, skills and other resources by local organizational partners. With LL, management support, governance, IT, and premises were donated in-kind by organizational members of the collaborative steering group. PIC also relied on donations from strategic partners including facilities. Strategic partners also provided social capital to PIC in the form of introductions to potential funders as well as free publicity which enabled PIC to establish its reputation in the community. Community members also contributed their time and energy supporting the organization, sometimes at own expense. Within LL, sub-groups of staff from organizational partners as well as community members were formed to lead specific initiatives without a fixed process or set of actions governing their contribution. In essence, community members were “co-owners” of the organization, sharing in benefits as well as responding to challenges. Two central premises framed these relationships and exchanges: the flexible and inclusive approach to asset identification and deployment enabled everyone to be contributors, while reciprocity allowed individual and organizational contributors also to receive benefits whether immediately or deferred. In the LL case, a virtuous cycle of access to more resources and improved understanding of community needs and aspirations has been initiated.

Lastly, the highlighted projects are in a position to move co-creation activities to a new level. For PIC new partnerships are in development, such as with local health centers. This could lead to new revenue-generating activities, for example expediting discharges from hospital, helping lower re-admission rates and maintaining good health through preventive measures. This is leading to the definition of a new kind of volunteer to support aging-in-place: volunteers that are specifically trained and available to work with older people and the medical professionals handling their cases.

### Currency Infrastructure

All the analyzed cases recognize the importance of creating opportunities for novel forms of transaction mediated by currencies other than regular (fiat) money. To varying degrees, they represent experiments in developing community (non-money) economies that operate alongside and as complements to the market (money) economy by supporting transactions aimed primarily at delivering social value. Local community economies can provide an overarching mechanism for building robust, inclusive and resilient communities because they provide opportunities for participation by all citizens and for contributions to be made in kind to own and community wellbeing and to addressing priority social challenges. They mobilize locally-available assets and spare capacities that would otherwise go to waste and put these to productive use to address the challenges communities face and to deliver positive social value.

Each of the case studies has identified one or more infrastructural elements—building blocks—for developing secondary social economies. Their innovations respond to the challenges they have faced. One such challenge is for new ways to stimulate citizen participation through schemes that recognize and reward contributions to own and community wellbeing through immediate and useful benefits, rather than only deferred and corresponding benefits. Just as there must be opportunities to earn credits, there must be opportunities to redeem credits. LL experiments with a solution that issues a membership card to those who give time to community projects and has developed a network of local businesses willing to offer discounts to card holders. A “spend” network is an element of secondary economy infrastructure that can leverage more local assets and spare capacities into productive use, including those of local businesses and anchor organizations, offering recognition to those who contribute to the community and rewarding them for their contributions.

There is also a need for a community currency that is not tied to the value of any regular fiat currency but can be used locally as a medium of exchange, a token of value and a unit of account. Traditionally, time banks use time as a community currency and hours of service as currency units. Traditionally, they use time banking software both as a platform for organizing service exchanges and for providing accounting functions. The case study initiatives have found these traditional arrangements inadequate on several counts. Time is less suitable as a unit of exchange and fixed values of time can be less appropriate in contexts where the concern is for outcomes and their importance and when transactions extend beyond only service exchanges to include goods and/or discounts on everyday purchases. Organizers at LL note that it is easier to attract citizens to give time to environmental projects than to care-in-community efforts. This suggests that more flexible arrangements involving negotiated values for contributions to the community might be needed—more of a “market” for “community credits”—rather than assuming fixed values for time inputs.

The case studies are all involved in experiments with innovative solutions they seek to develop within the framework of a community economy. They, therefore, all recognize the need for monitoring and evaluation as well as openness and transparency of data and information as a basis for (social) learning. These are needed to find what works and what does not and to fine tune the designs of promising models. A challenge is that when the community economy has a scope beyond that of a time bank, time banking software is no longer a sufficient mechanism for data collection. For this reason, in two of the cases, HERMAN and LL, there is recognition of the need for a digital community currency, so that the community economy and its development can be modeled, transactions can be tracked, and the behaviors of community economy participants better understood.

Another need is for an interface between the non-money community economy and the regular money economy in order to enable community organizations to secure an income stream to cover the money costs they face. While not necessarily large in absolute terms, these are nevertheless crucial to cover if the initiatives are to be sustained. This issue interfaces with the need for a digital community currency since digitization offers routes to income streams. Service commissioners may be willing to pay to secure wanted outcomes but need verification of performance and delivery. Digital currency provides a way to track transactions and verify activities. In the process, it also provides a degree of safeguarding since digitization provides a record of the transaction, what it involves, the parties, the time, and the place. The more able schemes are to assure commissioners and funders that targeted outcomes are being achieved, the more fundable the schemes become.

Furthermore, the data gathered by a digital currency as it moves from one electronic wallet/account to another constitutes a new community-generated asset, since the data can be valorized as economic and social intelligence and as a valuable support to business and individual stakeholders, for example, in discharging and reporting corporate social responsibility and verifying corporate community engagement. There is a potential for big data applications, for example, in support of research into the health and well-being benefits of active citizenship. At the individual level, transaction records can contribute to CVs, demonstrating community service and experience. Business plans for valorizing data and generating income to return to the community organizations can be part of their sustainability strategies.

Distributed ledgers offer new ways to secure, store, verify and query transaction data and to avoid escalating server costs as the number of transactions in a community economy grows. For this reason, block-chain solutions may offer the best technology platform for the development of digital community currencies, avoiding the costs of cryptocurrency solutions but benefitting from the same distributed ledger, horizontal expansion, and flexible development possibilities.

### The Role and Participation of Government

Weaver and Marks (2017) identified three distinct pathways to financial growth and sustainability among studied social innovations. These include: an external funding pathway that involves seeking investment or income from establishment actors, such as service commissioners who provide funding but set conditions on this; an autonomous funding pathway through which a social innovation organization develops its own income stream to self-finance its activities and fund continuity and growth, typically through related social enterprise activity; and an embedded pathway whereby the social innovation organization partners with an existing organization and receives financial support from the larger (host) organization in return for helping it deliver its mission. Each pathway informs relationships with statutory authorities; for example, the external funding pathway necessitates that social innovation organizations accept a role of traditional government-contracted service provider with requirements to meet certain performance and accountability standards and, to a certain extent, to adapt operations to address statutorily-driven goals and objectives. This can result in a loss of autonomy and mission drift that social innovation organizations and their members may or may not be willing to accept, with implications for external and internal governance of initiatives (Weaver and Marks, 2017).

Interestingly, the four projects studied over time embraced aspects of autonomous funding with some embracing it as the dominant financing pathway. Projects required some funding to cover base-level operating expenses, and some sought this funding from statutory authorities or private sources. However, because traditional statutory funding has become increasingly uncertain or unsustainable, many of the projects sought to diversify funding and limit dependencies by working to generate their own income stream to support their operation. Examples ranged from the social enterprise boutique (PIC) to annual membership and activity fees paid by those receiving care services (G&TC). Interestingly, studies show that embracing the autonomous funding pathway is a common feature of time banks that have sustained over time (Weaver and Marks, 2017).

In some cases, the role of local and central government in supporting social innovation changed with less reliance on public funding. With PIC, for example, government entities were asked to provide entrée to private funders and to help legitimize PIC when it first started out. PIC also brought funding opportunities to government agencies for consideration, offering the in-kind services match as an enticement to partner. Similarly, with LL, government entities viewed LL as a “go-to” organization, seeking creative responses to difficult challenges with or without a direct offer of funding. With G&TC, the ability to attract significant numbers of users paying annual and activity-based fees makes them an attractive partner, pulling their own weight financially. It appears that with government entities, reciprocity often guided the relationship with the case study organizations. The ability of PIC to respond quickly to transport needs, for example, contributed to government officials providing the organizations with early notification of government funding for vans to transport non-ambulatory seniors. LL's responsiveness to needs helped secure a special relationship with other charities and local authorities.

Although social innovation organizations tend to choose a dominant funding pathway to achieve levels of sustainability, financial needs may vary over time and, with that, funding relations with traditional government funders may change. As social innovations mature, there is a pull to expand and scale operations which may require the building of organizational, managerial and technology capacities (Weaver et al., 2017). Recent emphasis on social impact investing, including using social impact bonds where private investors provide up-front funding for innovations and are reimbursed contingent on outcome attainment may provide new funding opportunities for social innovations if performance tracking systems are put in place (Marks and Weaver, 2017). Social innovation organizations may position themselves to compete for commissions as service providers, but in doing so need to evaluate the risk/rewards of participating in terms of mission, changing expectations of volunteers and relationship dynamics with statutory partners (Weaver et al., 2017).

### Creating Living Labs for Aging-in-Place

Systemic challenges facing the aging population require novel responses. The framework delineated here for experimenting and studying proposed solutions is one grounded in social innovation and specifically in community-led schemes, for example, grassroots activities that have the attribute to be place-based and largely bottom-up with a large and diverse group of local stakeholders. The proposed framework is a Living Lab for Aging-in-Place that serves as a methodology for experimentation and data collection as well as a social foundry for innovative services and products in support of aging. Through the review of the four cases it was learnt that a national strategy to implement this type of innovation is inadequate and does not match with local community identities (c.f. G&TC), that co-production had to include all social and demographic groups to generate multiple networks of value (c.f. HERMAN), that the involvement of businesses is fundamental in creating spending networks that enable immediate rewards for active citizens growing their participation (c.f. LL) and that reliance on philanthropic and action research grants is not sufficient and creative forms of co-finance are necessary (c.f. G&TC, PIC).

In essence the Living Lab for Aging-in-Place ought to include a complementary economy that enables the social and economic participation of those who are traditionally excluded due to lack of traditional currency and perceived societal irrelevance, for example aging and disabled individuals perceived by society at large as a financial drain and as unable to contribute. The development of the Living Lab for Aging-in-Place is underpinned by a digital infrastructure which serves the ability to track exchanges and collect and query data in real time. The functional modules of the framework are highlighted below:

- An open source digital infrastructure that is available to each Living Lab and that can be adapted to local needs. The open source of the digital solution will also enhance data integration if adopted in several Living Labs and will reduce the costs for each community as it will not require licensing. A distributed ledger has been identified as a suitable option as it keeps the cost of expansion to a minimum and enables multiple and distributed back up of the data to safeguard system integrity.
- Digital community currency that must be secure enough to enable trust in the transactions without overwhelming the users. The digital community currency ought to have no direct exchange rate with traditional currency in order to avoid the creation of illegal secondary markets and to reduce the perception of reward as a form of traditional payment.
- Community observatory function to inform the design and delivery of more inclusive products and services. This will attract the attention and interest of businesses and entrepreneurs willing to enhance their understanding of the aging population within the context they are part of to gather user requirements and pinpoint opportunities that may lead to novel products and services. Within the community observatory function, older adults would be considered as expert users for existing products and expert advisors when generating novel concepts. This function pivots around the transformation of aging from a condition imposing costs and symbolizing a deteriorating or decaying society to one representing assets and opportunities.
- Co-design and co-delivery methodology to create consensus around the strategic priorities of the place-based community and co-produce joined-up, inter-departmental and inter-organizational responses.
- The role of a community intermediary capable of professional coordination of grassroots local projects delivered against the codesigned community strategy and delivering added value to participants (givers and recipients). The intervention of the intermediary facilitates inter-departmental and inter-organizational synergies in tackling complex societal challenges as demonstrated in previous Living Lab experimentations (Almirall and Wareham, 2008). The intermediary also helps make community activities investment-ready; e.g., for Social Investment Bonds (SIBs) that have proved successful in addressing societal challenges in other domains, such as young offenders (Dermine, 2014).
- The participation of local businesses and organizations with spare capacities in providing opportunities to spend earned community currency locally, thereby encouraging active citizenship.
- Continuous interface with local and central policymakers to provide the evidence base for understanding and modeling impacts of social policy interventions and innovations.
- A new language in social innovation research where real communities are involved in capturing and communicating an emerging semantic spectrum. This may enable the emerging relationships between the community and active citizenship to be more clearly specified, for example, the idea of being commissioned rather than contracted to co-deliver services and for participation to be recognized and rewarded but not recompensed by a salary paid in fiat currency.

The list of the above components of a Living Lab for Aging-in-Place has been informed by primary and secondary research and represents the foundations upon which to build an ambitious programme of experimentation and research. This requires forward-looking, agile and untraditionally risk-taking communities, local authorities, funding bodies, and businesses. Current paradigms for seeking and implementing solutions have not been able to respond effectively to the challenge of transformative change that an aging population demonstrates is required. The Living Lab for Aging-in-Place is a methodology as well as a set of research assumptions that ought to be trialed in an agile environment and with the compassionate understanding that live experimentations can and should be amended as more evidence is collected about the needs and aspirations of the aging population and communities at large.

## Data Availability

The datasets generated for this study are available on request to the corresponding author.

## Author Contributions

All authors contributed to the paper in its entirety. Specifically, GS developed the abstract and introduction, wrote the LL and G&TC case studies, contributed to the methodology section and the section on the value model. She also led the conclusion section. PW led on the methodology and social innovation sections. He wrote the HERMAN case study and the section on currency infrastructure. MM wrote the PIC case study and the sections on co-creation and the roles of government. CV wrote the section on current social care models.

### Conflict of Interest Statement

The authors declare that the research was conducted in the absence of any commercial or financial relationships that could be construed as a potential conflict of interest.
